# *C. elegans* Hemidesmosomes Sense Collagen Damage to Trigger Innate Immune Response in the Epidermis

**DOI:** 10.3390/cells12182223

**Published:** 2023-09-06

**Authors:** Yi Zhu, Wenna Li, Yifang Dong, Chujie Xia, Rong Fu

**Affiliations:** Jiangsu Key Laboratory of Infection and Immunity, Institutes of Biology and Medical Sciences, Soochow University, Suzhou 215123, China; zhuyi216@suda.edu.cn (Y.Z.);

**Keywords:** epidermis, innate immunity, collagen, hemidesmosome, antimicrobial peptide

## Abstract

The collagens are an enormous family of extracellular matrix proteins that play dominant roles in cell adhesion, migration and tissue remodeling under many physiological and pathological conditions. However, their function mechanisms in regulating innate immunity remain largely undiscovered. Here we use *C. elegans* epidermis as the model to address this question. The *C. elegans* epidermis is covered with a collagen-rich cuticle exoskeleton and can produce antimicrobial peptides (AMPs) against invading pathogens or physical injury. Through an RNAi screen against collagen-encoding genes, we found that except the previously reported six DPY collagens and the BLI-1 collagen, the majority of collagens tested appear unable to trigger epidermal immune defense when damaged. Further investigation suggests that the six DPY collagens form a specific substructure, which regulates the interaction between BLI-1 and the hemidesmosome receptor MUP-4. The separation of BLI-1 with MUP-4 caused by collagen damage leads to the detachment of the STAT transcription factor-like protein STA-2 from hemidesmosomes and the induction of AMPs. Our findings uncover the mechanism how collagens are organized into a damage sensor and how the epidermis senses collagen damage to mount an immune defense.

## 1. Introduction

Extracellular matrix (ECM) is the material basis for connecting cells, endowing various tissues and organs with morphological structure and mechanical properties. ECM is mainly composed of collagens, fibronectin, laminins and proteoglycans. As the core structural proteins of ECM, collagens not only provide structural support to cells, but also participate in various biological processes such as proliferation, differentiation, migration and inflammation [1]. Increasing evidence has revealed a close interplay between collagens and the immune system. For example, cytokines and proteases secreted by infiltrating immune cells regulate the synthesis and cleavage of collagens [2,3,4]. In turn, the abnormal expression and bioactive fragments of collagens affect inflammatory response by regulating the chemotaxis, activity and differentiation of immune cells [4,5,6,7,8]. Most studies focus on the effect of a single collagen component on specific immune cells. However, the regulation of the collagen network on the innate immune response of epithelial cells remains largely unknown.

The *C. elegans* epidermis is an ideal model to study the regulation mechanism of innate immunity by collagens. The epidermis of *C. elegans* adult is mainly composed of a syncytium termed hyp7, which covers most of the body [9]. The *C. elegans* hemidesmosomes (CeHDs) are trans-epidermal attachment structure composed of the apical and basal units connected by intermediate filaments (IFs). Apical CeHDs connect the cuticle through the transmembrane receptor MUP-4, while basal CeHDs link the basement ECM and body wall muscles through the receptor LET-805 [10,11,12]. The induction of antimicrobial peptides (AMPs) is an important innate immune response of the *C. elegans* epidermis for resistance to pathogens and physical injury [13]. *C. elegans* AMPs include two related families: the neuropeptide-like proteins (NLPs) and the caenacins (CNCs) [13,14,15,16]. Fungal infection and wounding activate the G-protein-coupled receptor DCAR-1, which transmits signals to the conserved p38 mitogen-activated protein kinase (MAPK) signaling pathway [14,15,17]. This pathway acts downstream of the toll-interleukin-like adaptor TIR-1 and upstream of the STAT transcription factor-like protein STA-2, ultimately inducing the expression of NLPs [14,15,16,18,19]. Besides STA-2, the GATA transcription factor ELT-3 is also required for the *nlp* gene induction following infection [13,14]. A tyrosine metabolite, hydroxyphenyllactic acid (HPLA), was identified as the endogenous ligand of DCAR-1, which is increased after *D. coniospora* infection to activate DCAR-1 [14,15,17]. The activation of the *cnc-2* gene cluster also depends on the p38-MAPK pathway after injury. However, during infection, its activation is dependent on the neuron-derived TGF-β signaling pathway but not the p38-MAPK pathway [14,16,20]. *nlp-29* expression can also be elevated by hyperosmotic stress, which is mediated by the WNK-Ste20 pathway and the transcription factor ELT-3 [13,21]. In addition to the above-mentioned pathways, there is a straightforward strategy for the epidermis to sense structural damage. Upon damage of apical CeHDs, STA-2 binding to the CeHD receptor MUP-4 is released into the cytoplasm to drive AMP expression [14,22,23].

The *C. elegans* epidermis is predicted to synthesize and secrete more than 170 types of collagens for the formation of the cuticle, an apical ECM [24]. The cuticle encloses the worm to protect it from external insults and maintain its shape and movement [25,26,27,28,29,30]. Loss of specific cuticle collagens (six DPY collagens and the BLI-1 collagen) or cuticle replacement at molt triggers innate immune responses in the epidermis [13,17,22,31,32,33]. In addition, the physical disruption of the cuticle occurs upon fungal infection and mechanical wounding. These findings suggest that collagen damage caused by internal or external insults may be the first signal to activate innate immunity. However, it is still unknown whether more collagens are involved in the innate immune response, and how specific collagens are assembled into a damage sensor. Loss of the p38 MAPK PMK-1 partially inhibits the elevated expression of P*nlp-29*::GFP in *dpy-9* mutants [13]. Loss of *dcar-1* function reduces the induction of multiple *nlp* genes to varying degrees in the *dpy-10* mutant background [17]. Among *nlp* genes regulated by *dpy-10*, the activation of *nlp-28* and *nlp-29* is least dependent on *dcar-1* [17]. Since the DCAR-1-p38 pathway only partially accounts for the upregulation of *nlp-29* caused by collagen damage, how the signal of collagen damage is perceived and relayed to induce epidermal immune response remains obscure. 

Here, we utilized a large-scale RNAi screen to systemically investigate the involvement of collagens in the innate immune response of the epidermis. We found that most of the tested collagens do not seem to possess the previously reported immune-regulating function of the specific DPY collagens and the BLI-1 collagen. Further investigation suggests that the epidermis senses the damage of specific collagens through the apical CeHD receptor MUP-4, whose separation from the collagen ligand causes the release of STA-2 to activate immune defense. A specific DPY collagen substructure and BLI-1 (the presumptive collagen ligand of MUP-4) constitute a two-step cascade for damage sensing. Our study reveals a special function mechanism of the classical ligand-receptor interaction between collagens and adhesion structures in immune response of epithelial cells.

## 2. Materials and Methods

### 2.1. C. elegans Strains and Maintenance

*C. elegans* strains were maintained following standard procedures [34]. The worms were grown at 20°C and fed with OP50 *E. coli* unless noted otherwise. The strains carrying frls7[P*nlp-29*::GFP+P*col-12*::DsRed], *dpy-5(e907)*I;sEx15292[rCes C25A1.5::GFP+pCeh361], *dpy-17(e164)*III, *dcar-1(tm2484)*V, *tir-1(tm3036)*III, *dbl-1(nk3)*V, *sma-6(wk7)*II, *wnk-1(ok266)*IV, *elt-3(gk121)*X, *sta-2(ok1860)*V, *sek-1(km4)*X, *pmk-1(km25)*IV, *mup-4(mg36)*III;upIs1[*mup-4*::GFP+*rol-6(su1006)*], kals12[*col-19*::GFP] and *unc-119(ed3)*III;cgEx198 [(pJC14) *bli-1*::GFP + *unc-119(+)*] were obtained from the Caenorhabditis Genetics Center, which is funded by the National Institutes of Health Office of Research Infrastructure Programs (P40 OD010440). 

### 2.2. CRISPR/Cas9-Mediated Mutagenesis of the dpy-10 Gene

The CRISPR-Cas9 vector (pDD162-P*eft-3*::CAS9-PU6), Cas9-*dpy-10* sgRNA plasmid and protocol were kindly provided by the laboratory of Xiaochen Wang (IBP, Beijing, China). Mutant strains were generated by microinjecting 50 ng/μL Cas9-*dpy-10* sgRNA plasmid. The allele *sda6* with the mutation (a G to A substitution and a TTTCAACCTATA insertion in the second exon of *dpy-10*) was identified by dumpy phenotype and sequencing.

### 2.3. Molecular Biology and Transgenesis

All DNA fragments were amplified by PrimeSTAR GXL Premix (TaKaRa, Osaka, Japan). All fusion plasmids were constructed using the ClonExpress II One Step Cloning Kit or the ClonExpress MultiS One Step Cloning Kit (Vazyme Biotech, Nanjing, China). Translational mCherry fusions of the *dpy* genes (*dpy-2*, *dpy3*, *dpy-4*, *dpy-5* and *dpy-17*) were generated by cloning their corresponding genomic coding sequences plus 1600–2000 bp promoters into the pPD49.78 vector. The P*mup-4::mup-4*(extracellular domain deletion)*::gfp* plasmid was constructed by inserting a 5155 bp promoter and the coding sequence excluding the extracellular domain of the *mup-4* gene into the pPD95.75 vector. All plasmids were confirmed by sequencing before microinjection. All the plasmids above were injected into N2 at a concentration of 10 ng/µL, respectively. P*myo-2*::GFP or P*myo-3*::GFP was used as co-injection marker. The primer oligonucleotides utilized for constructing the plasmids in this study are listed in Table S1.

### 2.4. RNAi

The RNAi plasmids targeting genes (*dpy-2*, *dpy-9*, *dpy-10*, *dpy-13*, *wnk-1*, *gck-3*, *bli-6*, *rol-1*, *lon-3*, *col-20*, *col-73*, *col-168*, *col-178*, *col-180* and *col-181*) were generated by cloning 400–800 bp cDNA fragments into the L4440 vector. Other RNAi clones were obtained from the MRC RNAi library and verified by sequencing. The plates seeded with RNAi bacteria were made as previously described [35]. Strains were fed with RNAi bacteria from the L1 stage to the young adult stage together with control worms fed with HT115 *E. coli* containing the empty RNAi vector L4440.

### 2.5. Immunostaining and Fluorescence Microscopy

Young adults were fixed using the peroxide tube fixation protocol and stained by indirect immunofluorescence as previously described [36]. The STA-2 polyclonal antibody was raised by BML (Beijing, China) against peptides CRNLAPDEIYFDNQGAAT and CVAEEFQHKKSASAEGDW [22]. The MUP-4 polyclonal antibody was raised by Youke (Shanghai, China) against peptide PRAKLARPLYGDEMGDD as described previously [11]. The DPY-7 polyclonal antibody was raised by Youke (Shanghai, China) against peptide CPSSCGVQEIVAPSVSELDTNDEPEKPARGGYSGGGYGKK. The dilution factors for the primary antibodies are: GFP (DSHB-GFP-12A6), 1:100; STA-2, 1:25; MUP-4, 1:500; DPY-7, 1:100. Alexa Fluor^®^ 488- and Alexa Fluor^®^ 568- labeled secondary antibodies (Molecular Probes, Eugene, OR, USA) were used at 1:800 dilution. Live animals were immobilized by 1 mM levamisole in M9 buffer on 2% agarose pad. Confocal images were obtained using Zeiss LSM800 confocal microscope. All image quantifications were performed using the ImageJ software (the 1.47d version) (https://imagej.nih.gov/ij/, accessed on 26 June 2012).

### 2.6. Quantitative RT-PCR Analysis

L1 larval arrest was induced by hatching embryos in the absence of food after bleaching. For gene expression analysis of *nlp-29* and *cnc-2*, young adults were collected. To examine the temporal expression pattern of collagens, worms were harvested every 2 h after release from an L1 arrest until L2 stage. Total RNA was extracted using the RNAiso plus reagent (TakaRa, Osaka, Japan) and reverse transcribed by primescript RT master mix (TakaRa, Osaka, Japan). Real-time PCR was performed using Faststart universal SYBR Green master (Roche, Basel, Switzerland) on Lightcycler 96 system (Roche, Basel, Switzerland). The expression levels of target genes were normalized using the reference gene *act-1*. Primers used in QPCR reactions are listed in Table S1.

### 2.7. Statistical Analysis

Statistical analysis was performed using GraphPad Prism 5.0 software. Data normality was verified by using the Shapiro–Wilk normality test. A two-tailed unpaired *t* test was used to calculate statistical significance, as described in the figure legends.

## 3. Results

### 3.1. Only a Small Group of Collagens Induces AMP Expression upon Damage

**Figure 1:**
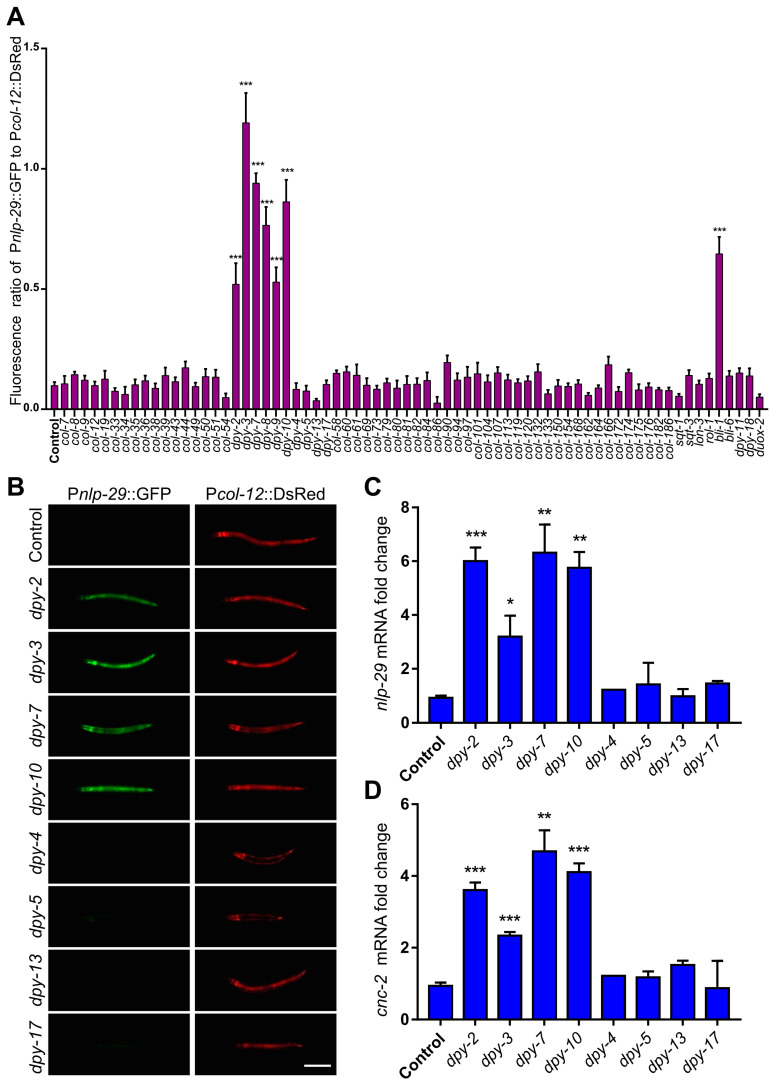
A small portion of collagens is responsible for AMP induction. (**A**) The fluorescence ratio of P*nlp-29*::GFP to P*col-12*::DsRed in young adults lacking different collagens or collagen modifying enzymes (*e907* and *e164* mutation for inactivating *dpy-5* and *dpy-17*, RNAi for silencing other genes) compared with control worms fed with L4440 (the empty RNAi vector). (**B**) Expression of P*nlp-29*::GFP in young adults with damage of different DPY collagen proteins compared with control worms fed with L4440. P*col-12*::DsRed served as an internal control. Scale bar, 200 μm. (**C**,**D**) Quantitative RT-PCR results show *nlp-29* or *cnc-2* expression in young adults with deletion of different DPY collagens compared with control worms fed with L4440. Error bars, mean ± SEM. *, *p* < 0.05; **, *p* < 0.01, ***, *p* < 0.001 (two-tailed unpaired *t* test).

In order to comprehensively analyze the regulation of collagens on innate immunity of the epidermis, we used RNAi to disrupt collagen proteins encoded by the DumPY (*dpy*, n = 10), COLlagen (*col*, n = 57), ROLler (*rol*, n = 1), BLIster (*bli*, n = 2), SQuaT (*sqt*, n = 2), and LONg (*lon*, n = 1) gene classes and three collagen modifying enzymes (*dpy-11*, *dpy-18* and *duox-2*) one by one. We then tested for induction of the antimicrobial peptide using the transcriptional GFP reporter of *nlp-29* in young adults. Consistent with previous reports, defects of any one of the seven collagens (DPY-2, DPY-3, DPY-7, DPY-8, DPY-9, DPY-10 and BLI-1) significantly enhances the fluorescence intensity of P*nlp-29*::GFP [13,17,22,31] (Figure 1A,B). Unexpectedly, loss of other tested collagens and collagen modifying factors did not activate the expression of P*nlp-29*::GFP (Figure 1A,B). QPCR results confirmed that loss of *dpy-2*, *dpy-3*, *dpy-7* and *dpy-10* upregulated the expression level of *nlp-29*, whereas inactivation of *dpy-4*, *dpy-5*, *dpy-13* and *dpy-17* did not affect *nlp-29* expression (Figure 1C). In addition, another AMP gene, *cnc-2*, was also transcriptionally upregulated under RNAi treatment against *dpy-2*, *dpy-3*, *dpy-7* or *dpy-10* but not *dpy-4*, *dpy-5*, *dpy-13* or *dpy-17* (Figure 1D). No more collagen components were found to activate the immune response when damaged, suggesting that the seven collagens may be the core cuticle components for immune regulation.

### 3.2. The Immune Response upon Collagen Loss Requires the Activation of STA-2 by Non-Classical Signaling Pathways

**Figure 2:**
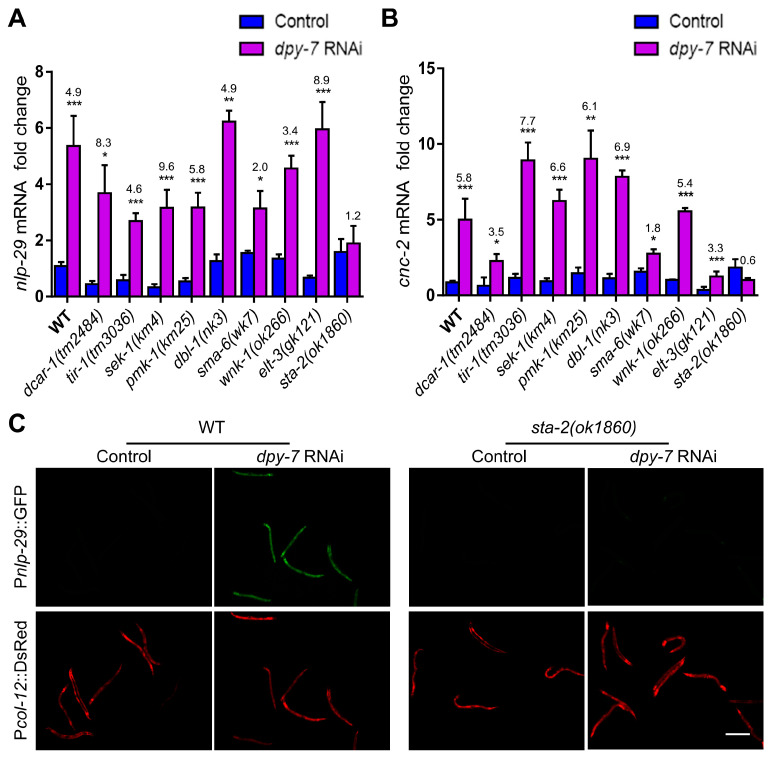
AMP induction by collagen damage is dependent on STA-2 activation by non-classical signaling pathways. (**A**,**B**) Quantitative RT-PCR results show fold induction (*dpy-7* vs. WT, *dpy-7*; mutation vs. mutation) of *nlp-29* or *cnc-2* after *dpy-7* RNAi treatment in wild-type (WT) young adults and loss-of-function mutants of *dcar-1*, *tir-1*, *sek-1*, *pmk-1*, *dbl-1*, *sma-6*, *wnk-1*, *elt-3* and *sta-2*. Error bars, mean ± SEM. *, *p* < 0.05; **, *p* < 0.01, ***, *p* < 0.001 (two-tailed unpaired *t* test). (**C**) Fluorescence images show the expression of P*nlp-29*::GFP in the wild-type and *sta-2(ok1860)* young adults treated with L4440 (control) or *dpy-7* RNAi. P*col-12*::DsRed served as an internal control. Scale bar, 400 μm.

To investigate the downstream mechanism by which cuticle collagens regulate AMP induction in the epidermis, we examined immune-related signaling molecules involved in fungal infection, physical injury, or hyperosmotic stress. As shown in the QPCR analysis of Figure 2A,B, *dpy-7* RNAi still up-regulated the expression of *nlp-29* and *cnc-2* to varying degrees in the loss-of-function mutants of G protein-coupled receptor DCAR-1, Toll-interleukin 1 receptor (TIR) domain adaptor TIR-1, SEK-1 and PMK-1 of the p38-MAPK pathway, DBL-1 and SMA-6 of the TGF-β pathway, WNK-type protein kinase WNK-1, or the transcription factor ELT-3 (Figure 2A,B). Only the loss of STA-2 blocked the elevation of AMP transcription caused by *dpy-7* inactivation (Figure 2A,B). Consistent with the QPCR results, *dpy-7* RNAi failed to enhance the intensity of P*nlp-29*::GFP in *sta-2* mutants (Figure 2C). Considering that the loss of the DPY collagens with immune regulatory function increases permeability and activates osmotic stress responses of the epidermis [25,31,37,38], we further verified whether the osmotic-pressure-related signaling pathway is implicated in immune regulation by collagens. Akin to the results using *dpy-7* RNAi, AMP induction in *dpy-10* mutants was not blocked by the inactivation of *wnk-1* or *gck-3* (Figure S1), both of which are required for AMP induction by hyperosmotic stress [21]. This result confirmed that the upregulation of *nlp-29* by collagen deficiency is not attributed to the intrinsic alteration of osmotic homeostasis. Taken together, these results suggest that the induction of innate immune response by collagen damage depends on the activation of STA-2 by non-classical signaling pathways.

### 3.3. Specific DPY Collagens Regulate the Innate Immune Response through the MUP-4/STA-2 Complex

**Figure 3:**
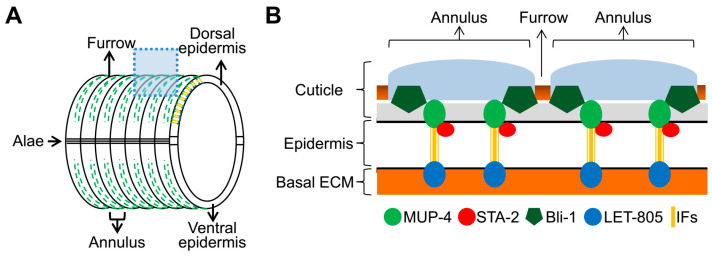

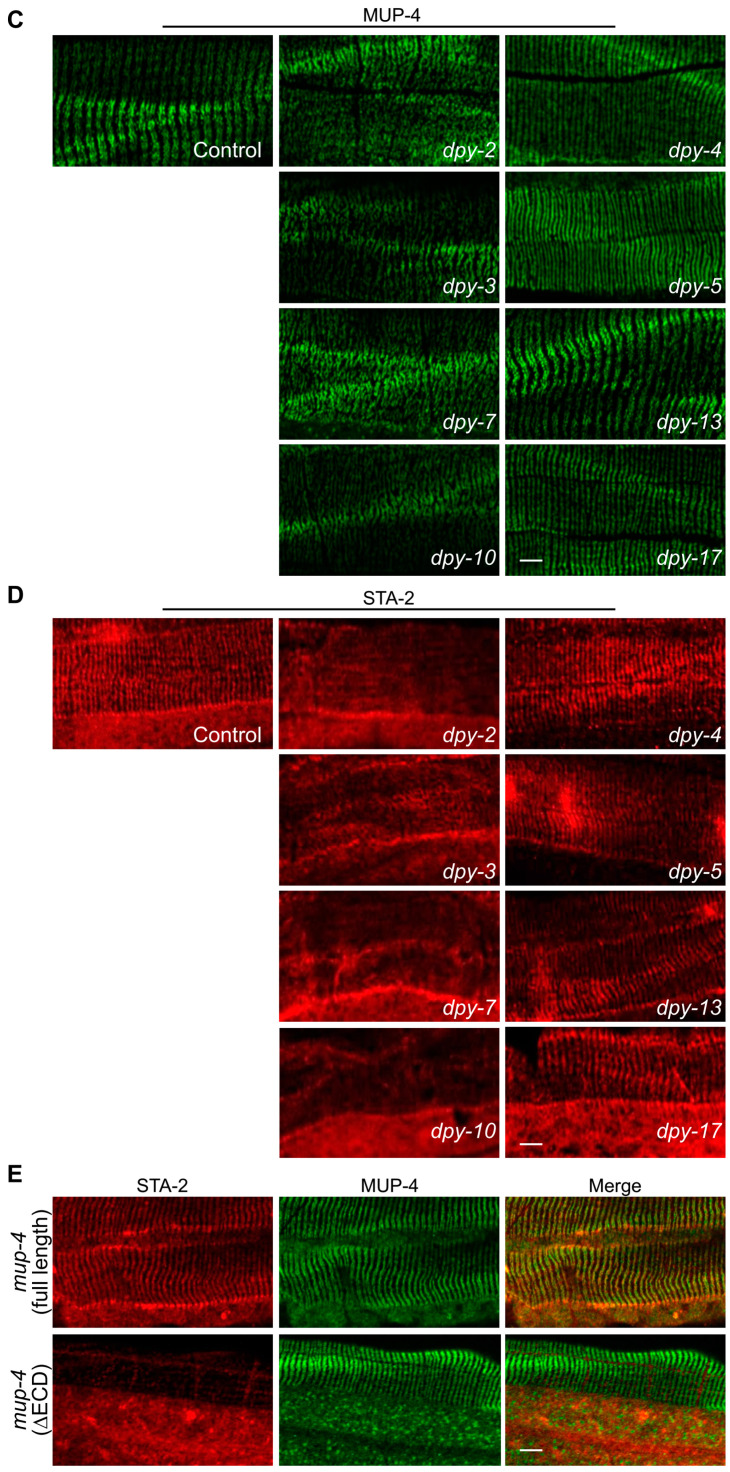
MUP-4/STA-2 complex is disrupted by damage of DPY collagens that elicits innate immune response. (**A**) Diagram of the mid-body region of a *C. elegans* adult showing cuticular structures (annulus, furrow and alae) and the underlying hyp7 epidermis. The dotted box represents the position for the longitudinal cross section of the epidermis in (**B**). Dotted green lines represent apical CeHDs. (**B**) Diagram of a longitudinal cross section through the cuticle and epidermis, indicating the localization and some key components of CeHDs. The apical CeHD receptor MUP-4 connects the cuticle, while the basal CeHD receptor LET-805 connects the basal ECM. The apical and basal CeHD units are linked through intermediate filaments (IFs). Direct physical interaction between MUP-4 and STA-2 has been confirmed by a previous reported co-IP experiment. (**C**,**D**) Representative confocal images of MUP-4 (**C**) and STA-2 (**D**) immunostaining in young adults with damaged collagens by RNAi against *dpy-2*, *dpy-3*, *dpy-4*, *dpy-7*, *dpy-10* or *dpy-13*, or loss-of-function mutations of *dpy-5* or *dpy-17* compared with control worms fed with L4440. (**E**) Immunostaining of MUP-4 (green) and STA-2 (red) in young adults with full-length and truncated (extracellular domain deletion/∆ECD) *mup-4::gfp* fusions. Scale bars, 10 μm.

The DPY-7 collagen modulates innate immune responses in a manner reminiscent of the hemidesmosome receptor MUP-4, whose disruption also provokes AMP transcription via STA-2 but not other immune signaling pathways. MUP-4 exerts immune surveillance function through binding and sequestering STA-2 under normal conditions and releasing STA-2 to induce AMPs upon apical structural damage [22]. Since MUP-4 connects the cuticle with the epidermis and contains a collagen binding domain in its extracellular portion [11], we speculate that cuticle collagens may regulate the innate immune response of epidermal cells through the MUP-4/STA-2 complex. To verify this hypothesis, we examined the effect of collagen deficiency on the localization of MUP-4 and STA-2 proteins in young adults by immunostaining. The apical CeHD receptor MUP-4 was organized into paralleled stripes in wild-type worms (Figure 3A–C). The silencing of *dpy-2*, *dpy-3*, *dpy-7* and *dpy-10* that initiates innate immune response caused a complete loss of parallel MUP-4 stripes (Figure 3C). In contrast, the striped pattern of MUP-4 was not significantly affected by the inactivation of *dpy-4*, *dpy-5*, *dpy-13* or *dpy-17* that was unable to induce AMP production (Figure 3C). In accordance with the effect of collagens on MUP-4, RNAi against *dpy-2*, *dpy-3*, *dpy-7* or *dpy-10* disturbed the CeHD-like pattern of STA-2, whereas DPY-4, DPY-5, DPY-13 and DPY-17 were dispensable for the CeHD localization of STA-2 (Figure 3D). Moreover, the deletion of the extracellular portion of MUP-4 containing the collagen-binding domain caused STA-2 detachment from CeHDs, suggesting that the interaction between MUP-4 and STA-2 responds to signals from ECM collagens (Figure 3E). These results support the notion that the damage of specific DPY collagens disassociates the MUP-4/STA-2 complex to activate STA-2, thereby inducing an immune response.

### 3.4. The Immunomodulatory Function of DPY Collagens Correlates with Their Expression Time but Not Their Localization

**Figure 4:**
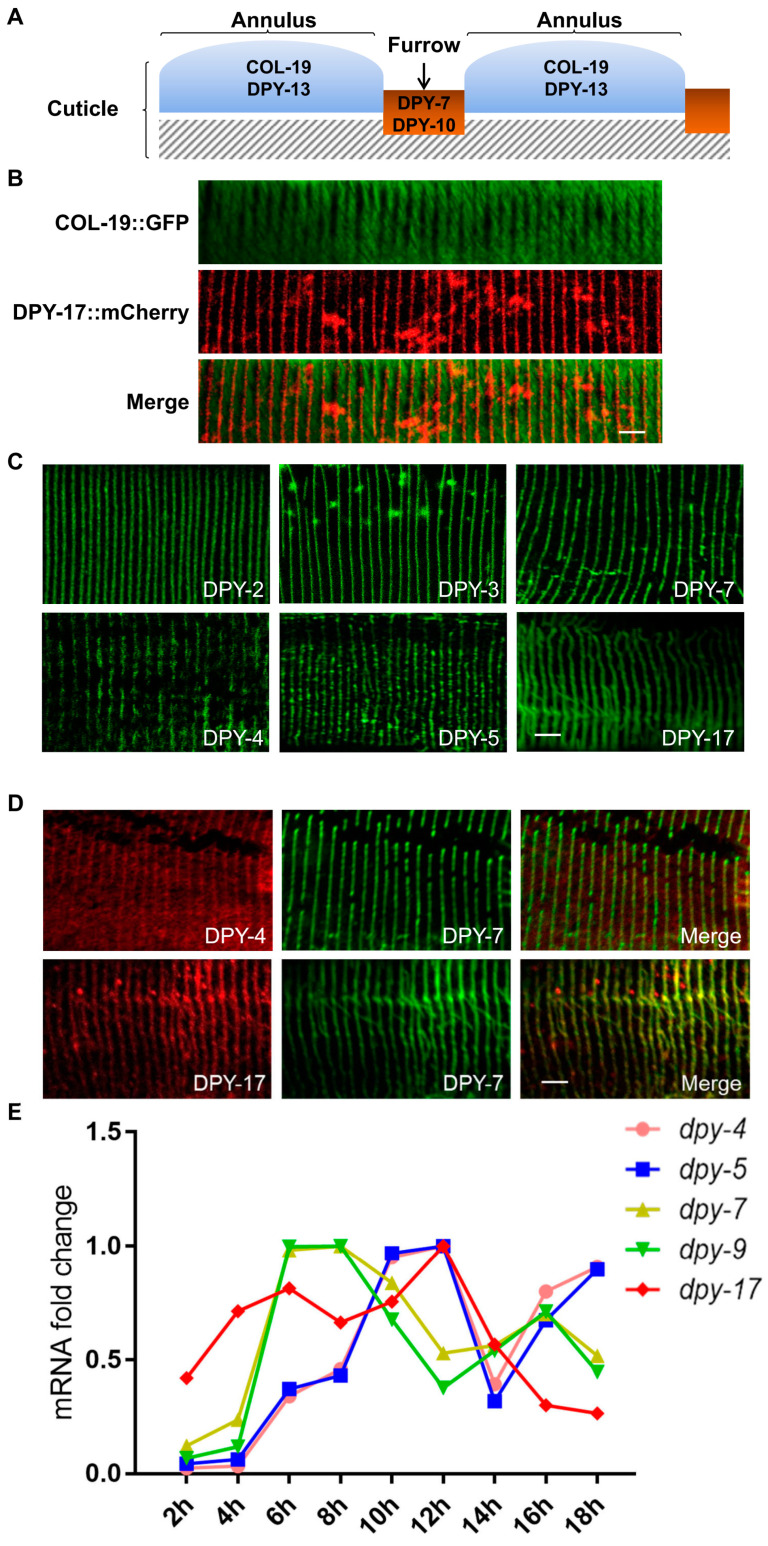
DPY collagens with immunomodulatory function are located in furrows and expressed early. (**A**) Diagram of known collagen localization. DPY-7 and DPY-10 are in furrows, while COL-19 and DPY-13 are in annuli. (**B**) The patterns and relative positions of the annulus collagen COL-19 (green, broad bands) and the furrow collagen DPY-17 (red, narrow bands) in a young adult. Scale bars, 10 μm. (**C**) Localization patterns of collagens labeled by DPY-2::mCherry, DPY-3::mCherry, DPY-4::mCherry, DPY-5::mCherry, DPY-17::mCherry or anti-DPY-7 immunostaining in young adults. Scale bars, 10 μm. (**D**) Colocalization of DPY-4 and DPY-17 (red, collagens without AMP induction ability) with DPY-7 (green, the collagen with AMP induction ability) in young adults. Scale bars, 10 μm. (**E**) Quantitative RT-PCR results show temporal changes in mRNA abundance for five different *dpy* collagen genes from L1 to L2 stage.

We next explored how specific collagens are organized into a damage sensor to activate innate immunity. Given that immunomodulatory collagens can impact the localization of MUP-4 (Figure 3C) [22,39], we firstly asked whether the immunomodulatory function of collagens correlates with their specific localization. The cuticle collagens generally exhibit as narrow stripes and broad stripes, corresponding to circumferential furrows and annuli, respectively (Figure 4A,B) [24]. It is known that DPY-7 and DPY-10 are located within furrows, while COL-19 and DPY-13 are located in annuli (Figure 4A,B) [40,41]. Thus, it is tempting to speculate that furrow collagens are responsible for immune regulation. To clarify the localization pattern of DPY-2, DPY-3, DPY-4, DPY-5 and DPY-17, we constructed transgenic strains expressing their mCherry fusion proteins. Unexpectedly, all of these collagens were displayed as narrow stripes similar to DPY-7 in young adults, suggesting that they are all located within furrows (Figure 4C). Double-labeling experiments showed that DPY-17 stripes were only positioned between the broad stripes of COL-19, and DPY-4 and DPY-17 were indeed co-localized with DPY-7 (Figure 4B,D). However, damage of DPY-4, DPY-5 or DPY-17 did not elicit an innate immune response like the insult of DPY-7 (Figure 1). It was previously found that formation of furrows requires DPY-7 and DPY-10 but not DPY-5 [31,40]. These findings reveal that not all furrow collagens affect immune responses, possibly because some of them are not essential for the formation of furrow structures.

The cuticle is synthesized prior to molting at the end of each larval stage. During each cuticle synthetic period, the cuticle collagen genes are expressed in early, intermediate, or late temporal series [24]. The *dpy-2*, *dpy-3*, *dpy-7*, *dpy-8* and *dpy-10* genes are all early-expressed, whereas the *dpy-5* and *dpy-13* genes are intermediate-expressed [40]. The expression rhythm of collagens is apparently correlated with their ability to regulate innate immunity. To test this hypothesis, we examined the temporal mRNA expression of *dpy-4*, *dpy-9* and *dpy-17*, whose expression rhythms have not been determined. QPCR results showed that the expression time of *dpy-9* was synchronized with *dpy-7*, whereas *dpy-4* was temporally co-expressed with *dpy-5* (Figure 4E). Although the expression rhythm of *dpy-17* was different from other collagen genes, its mRNA abundance peaked concurrently with *dpy-5* (Figure 4E). Taken together, our experimental results and previous reports revealed that early-expressed DPY collagens can induce AMP production once absent, whereas the intermediate-expressed DPY collagens cannot.

### 3.5. The Six DPY Collagens form a Substructure to Conjointly Control Cuticle Assemly and Immune Response

**Figure 5:**
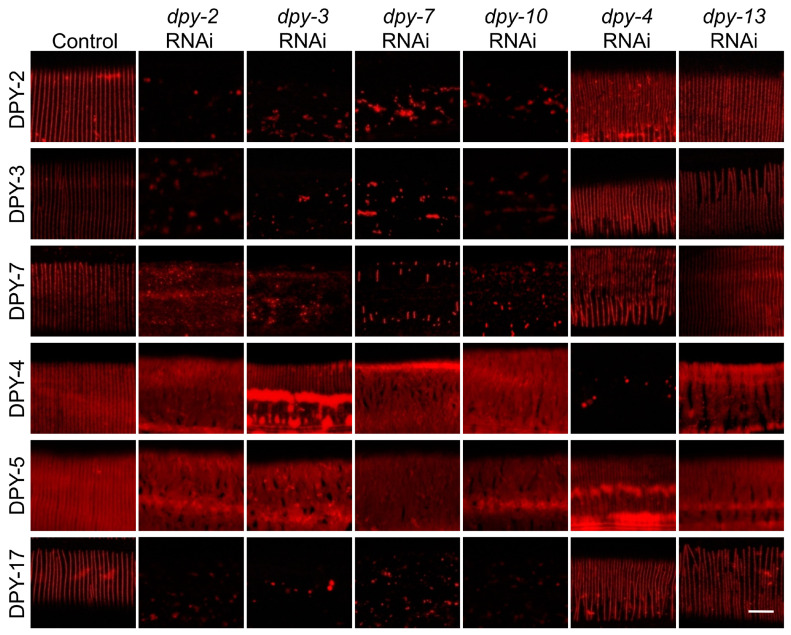
DPY collagens with immunomodulatory function cooperate to determine the cuticle localization of other collagens. Localization patterns of collagens labeled by DPY-2::mCherry, DPY-3::mCherry, DPY-4::mCherry, DPY-5::mCherry, DPY-17::mCherry or anti-DPY-7 immunostaining in young adults treated with the empty vector L4440 (control) or RNAi against different collagens. Scale bar, 10 μm.

We next asked whether the six DPY collagens function cooperatively as a unit to govern innate immune response. To verify this hypothesis, we systematically examined the influence of DPY collagens on each other’s localization in young adults. The results showed that knockdown of *dpy-2*, *dpy-3*, *dpy-7* or *dpy-10* led to a remarkable decrease in DPY-2, DPY-3, DPY-7 and DPY-17 in the cuticle, and dramatic disruption of the stripe pattern of DPY-4 and DPY-5 (Figure 5). Conversely, loss of *dpy-4* or *dpy-13* interfered with the band formation of DPY-4 and DPY-5 but not DPY-2, DPY-3, DPY-7 or DPY-17 (Figure 5). These results suggest that these early-expressed DPY collagens are interdependent and form a specific substructure to guide the arrangement of other collagens, thereby controlling the immune response of the epidermis. 

### 3.6. BLI-1/MUP-4, a Presumptive Collagen-Receptor Complex, Perceives the Damage Signal of DPY Collagens to Activate Immune Response

**Figure 6:**
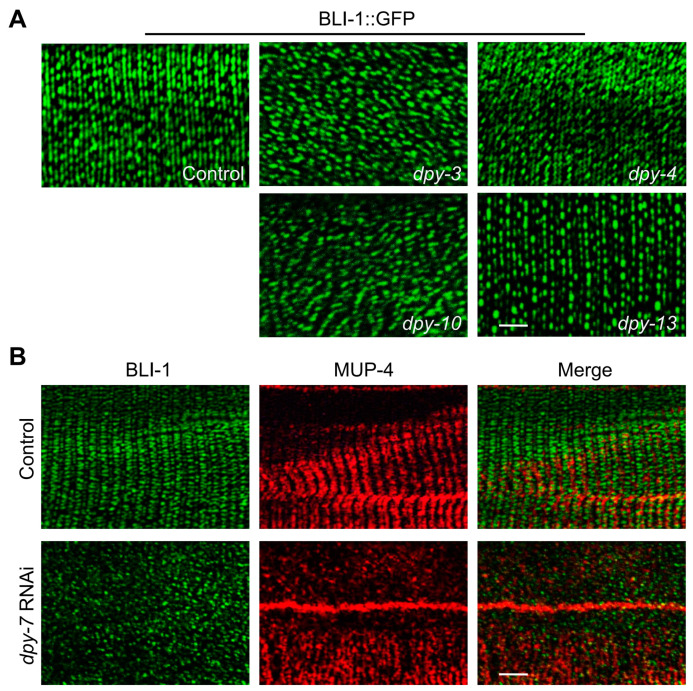
Damage of DPY collagens drives AMP expression via disrupting the interaction between BLI-1 and MUP-4. (**A**) Representative confocal images of BLI-1::GFP in young adults treated with the empty vector L4440 (control), RNAi with AMP induction phenotype (*dpy-3*, *dpy-10*) or RNAi without AMP induction phenotype (*dpy-4*, *dpy-13*). (**B**) Immunostaining of BLI-1::GFP (green) and MUP-4 (red) in *dpy-7* RNAi-treated young adults compared with empty vector L4440 (control) treated control animals. Scale bars, 10 μm.

A previous study showed that DPY-2-labeled stripes are positioned in between but not overlapping CeHD stripes, indicating that DPY collagens do not act as direct ligands for MUP-4 to exert immune regulatory effects [39]. We next asked which cuticle component is responsible for the direct interaction with apical CeHDs in the immune regulation process. The destruction of the collagen BLI-1 elevates the expression of AMPs by disintegrating the MUP-4/STA-2 complex [22]. Moreover, its localization partly overlaps with MUP-4 stripes [22]. These findings makes BLI-1 the candidate collagen protein capable of mediating AMP induction through directly interacting with MUP-4. To determine whether BLI-1 is the missing link between DPY collagens and MUP-4 in the immune regulation process, we firstly examined the effect of DPY collagens on BLI-1 localization. The results showed that BLI-1 assumed a dotted line pattern in wild-type young adults, which was completely abolished by the AMP-inducing collagen damage (inactivation of *dpy-3* or *dpy-10*) (Figure 6A). In contrast, the loss of *dpy-4* and *dpy-13* did not interfere with the periodic pattern of BLI-1, and as a result, no apparent AMP upregulation was detected (Figure 6A). We further explored the localization relationship between BLI-1 and MUP-4. The immunostaining of young adults showed that the spatial localization of BLI-1 and MUP-4 in CeHD regions was partly overlapped, suggesting a direct interaction between them (Figure 6B). Furthermore, we found that damage of DPY collagens with immune regulatory function resulted in remarkable separation of BLI-1 from MUP-4 (Figure 6B). In summary, we speculate that the specific substructure of DPY collagens modulates the innate immune response by affecting the interaction of BLI-1 with the immunoregulatory receptor MUP-4.

## 4. Conclusions and Discussion

In this study, we found through an extensive RNAi screen that the damage of most collagen proteins in the cuticle seems not to activate the innate immune response of the *C. elegans* epidermis. Only a few specific DPY collagens that are expressed at the same time and essential for the regular arrangement of other collagen proteins regulate innate immunity through the BLI-1 collagen. The downstream pathway analysis suggests that the damage of specific DPY collagens disrupts the interaction between the core collagen BLI-1 and its hemidesmosome receptor MUP-4, which in turn leads to the detachment and activation of the STAT-like transcription factor STA-2 and induction of AMPs. Taken together, we propose that ECM collagens might monitor the extracellular microenvironment through a two-step cascade model. One presumptive collagen-receptor complex (BLI-1/MUP-4) works as the core element to activate the immune response. To ensure sensitivity, another 6 DPY collagens regulate the binding of the core collagen and its receptor. Meanwhile, in order to prevent false alarms, the majority of collagens may be not involved in the regulation of immune response. Our findings uncover an elaborate and accurate mechanism for the collagens to detect tissue damage and activate immune defense, and provide a new train of thought for exploring the function mechanism of collagens in the pathogenesis of inflammatory diseases involving the structural damage of epithelial cells. 

Previous studies believed that the upregulation of *nlp-29* by collagen damage in *dpy-9* and *dpy-10* mutants was partly dependent on DCAR-1 and PMK-1 [13,17]. In contrast, our results showed that the induction of *nlp-29* by *dpy-7* RNAi was not inhibited by the loss of *dcar-1* or *pmk-1*. The possible reasons why our results are at odds with previous reports are as follows: firstly, the analytical output of previous reports is based on the comparison of *nlp-29* expression between mutants: *dpy*; *pmk-1* vs. *dpy* and *dpy*; *dcar-1* vs. *dpy*. We also observed that the expression level of *nlp-29* in *pmk-1* or *dcar-1* mutants was lower than that in wild-type worms upon *dpy-7* RNAi. However, our QPCR results showed that both *dcar-1* and *pmk-1* mutations lowered the basal level of *nlp-29* expression (Figure 2A), which may be difficult to be detected using the P*nlp-29*::GFP reporter. After normalization with *dcar-1* or *pmk-1* mutants, the fold-induction of *nlp-29* in *dpy-7*; *pmk-1* or *dpy-7*; *dcar-1* mutants (*dpy-7*; *pmk-1* vs. *pmk* and *dpy-7*; *dcar-1* vs. *dcar-1*) was actually not lower than that in wild-type worms with *dpy-7* RNAi treatment (*dpy-7* vs. WT) (Figure 2A). Therefore, we believe that the AMP induction by loss of DPY-7 is independent of DCAR-1 and the p38-MAPK cascade. That is to say, normalization may lead to a different interpretation of *dpy-9* and *dpy-10* data from previous studies. Notably, the deletion of *dpy-10* leads to an upregulation of the DCAR-1 ligand HPLA [17], suggesting that DPY-10 could indeed regulate immune response through the DCAR-1/p38-MAPK pathway. This raises an interesting possibility that although these six DPY collagens with immune regulatory functions behave in the same way in many aspects, they may still have some subtle differences in relaying damage-induced immune response. 

It is worth noting that the stiffness of the cuticle is lowered in *dpy* mutants that elicit immune response (*dpy-2*, *dpy-3*, *dpy-7* and *dpy-8*) but not in *dpy-13* mutants without AMP upregulation [42]. This raises an interesting hypothesis that the six DPY collagens may control downstream immune responses by providing additional mechanical signals to CeHDs, not just by impacting the interaction between CeHDs and their collagen ligands. In support of this hypothesis, CeHDs can sense the tension of basal muscle contraction and mediate mechanical signaling pathways [43,44]. The CeHDs exist simultaneously in the apical and basal membranes of the epidermis. It is an interesting scientific question whether and how the CeHD-associated STA-2 is regulated by mechanical signals from apical collagens. It is reasonable to presume that the biomechanical properties of hemidesmosomes allow them to act as a conserved pattern recognition receptor of collagen damage and other structural damage.

It is widely accepted that innate immune responses caused by the structural damage of cells contribute to tissue repair and regeneration, as well as numerous inflammatory diseases [45]. Accumulating evidence shows that the production and release of various endogenous molecules, termed damage-associated molecular patterns (DAMPs), promote sterile inflammation [45]. However, the current mainstream research focuses too much on the search for the production of new substances caused by damage (which can be called the additive model), and ignores that the loss of cellular structural integrity and homeostasis in the microenvironment can also serve as danger signals to activate immune response (which can be called subtraction model). Our study suggests that the disruption of the cellular structure and associated microenvironment (extracellular matrix, mechanical tension, etc.) caused by physical damage is likely to be the first signal to activate innate immune responses, which requires more extensive attention.

### Limitations of the Study

Our RNAi screen against *C. elegans* collagen-encoding genes shows that the RNAi of most collagen-related genes did not induce the expression of the AMP gene *nlp-29* in the *C. elegans* epidermis (Figure 1A). However, this observation does not necessarily confirm that these collagen-related genes do not have immune-regulating functions. Firstly, although we determine whether gene knockdown is effective by observing the morphological and developmental phenotype of RNAi, there are indeed some RNAis without visible phenotypes. We could not rule out the possibility that these RNAis did not alter the *nlp-29* expression due to inefficient gene knockdowns. Secondarily, our RNAi screen only examined one AMP type in animals under a limited set of age condition. It is still possible that these collagens regulate other immune effectors in a different development stage.

In this study, we speculate that BLI-1 may directly interact with MUP-4 based on the fact that BLI-1 not only co-localizes with MUP-4 but also regulates the expression of AMPs through MUP-4. In addition, the DPY collagens with immune-regulating functions affected the co-localization between BLI-1 and MUP-4. However, the direct binding between BLI-1 and MUP-4 requires biochemical methods for further validation.

## Data Availability

The raw data presented in this study are available on request from the corresponding author.

## Supplementary Materials

The following supporting information can be downloaded at: https://www.mdpi.com/article/10.3390/cells12182223/s1, Figure S1: AMP induction by collagen damage is independent on osmotic-related pathways; Table S1: Primers for plasmid construction and QPCR.
