# Supplementary material for: C. elegans Hemidesmosomes Sense Collagen Damage to Trigger Innate Immune Response in the Epidermis
Source: Cells. 2023 Sep 6;12(18):2223. doi: 10.3390/cells12182223 (PMC10526450; doi:10.3390/cells12182223)
Supplement: Supplementary file 1 [file cells-12-02223-s001.zip › cells-2556087-supplementary.pdf]

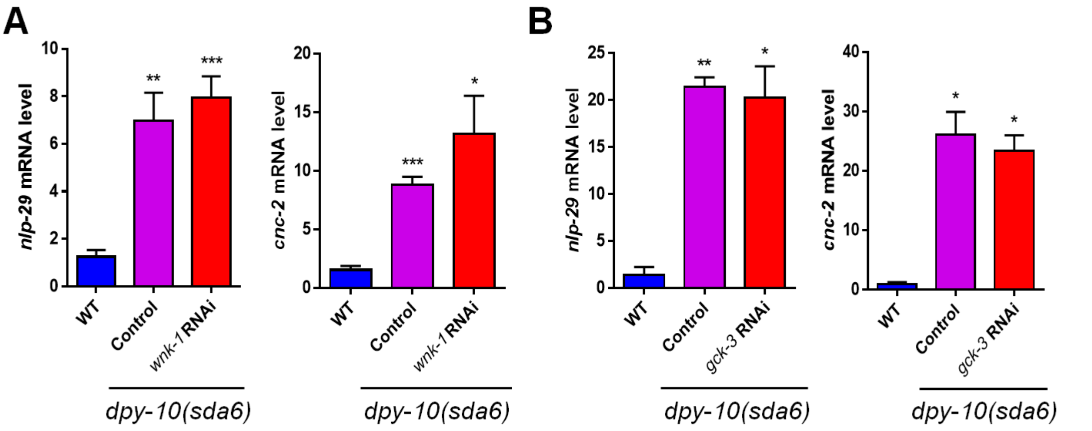

Figure S1. AMP induction by collagen damage is independent on osmotic-related pathways

(A-B) Quantitative RT-PCR results show *nlp-29* or *cnc-2* expression in *dpy-10(sda6)* with or without *wnk-1* RNAi (A) or *gck-3* RNAi (B) treatment compared with the wild-type control. Error bars, mean ± SEM. *, $p < 0.05$; **, $p < 0.01$, ***, $p < 0.001$ (two-tailed unpaired t test).

Table S1. Primers for plasmid construction and QPCR:

Plasmid	Forward primer (5'-> 3')	Reverse primer (5'-> 3')
pHMZ56[*dpy-2* RNAi]<br>For cloning *dpy-2* cDNA	attcgatatcaagcttgaaaagggctg<br>ctggatatg	cggtatcgataagcttcatttccgt<br>atccgtcgttt
pHMZ202[*dpy-9* RNAi]<br>For cloning *dpy-9* cDNA	attcgatatcaagctttatggagcttca<br>gggtcagg	cggtatcgataagctttggtggtg<br>ctccattcaata
pHMZ58[*dpy-10* RNAi]<br>For cloning *dpy-10* cDNA	attcgatatcaagctttcattgtctgtgtt<br>gctctcc	cggtatcgataagctttggtcctg<br>gattgatcaaaag
pHMZ36[*dpy-13* RNAi]<br>For cloning *dpy-13* cDNA	attcgatatcaagcttggatccatgaag<br>gacgtctg	cggtatcgataagcttgtttggtcc<br>ctttggtcctt
pHMZ164[*wnk-1* RNAi]<br>For cloning *wnk-1* cDNA	attcgatatcaagcttggagatcttgga<br>ttggcaac	cggtatcgataagcttttctgaacg<br>catccactttg
pHMZ165[*gck-3* RNAi]<br>For cloning *gck-3* cDNA	attcgatatcaagcttgaaaagaacag<br>gcgcaattc	cggtatcgataagcttcaatcgcg<br>aatcagtgtctt
pHMZ184[*col-181* RNAi]<br>For cloning *col-181* cDNA	attcgatatcaagcttccacggtgtcta<br>ccctcact	cggtatcgataagctttccgtctgg<br>tccattctctc
pHMZ183[*col-180* RNAi]<br>For cloning *col-180* cDNA	attcgatatcaagcttgtttctttcaccgc<br>cacatt	cggtatcgataagcttcttgggat<br>cctggatttcct
pHMZ166[*col-178* RNAi]<br>For cloning *col-178* cDNA	attcgatatcaagcttcagaagcgtcaa<br>actccaca	cggtatcgataagcttgtcctttcct<br>ggctctcctt
pHMZ176[*col-168* RNAi]<br>For cloning *col-168* cDNA	attcgatatcaagctttgaaggatcttcc<br>gttggac	cggtatcgataagcttatatcctgg<br>tgcggttcttg
pHMZ177[*col-73* RNAi]<br>For cloning *col-73* cDNA	attcgatatcaagcttccgtactcgttcc<br>gactctc	cggtatcgataagcttgtcctttcct<br>ggctctcca
pHMZ180[*col-20* RNAi]<br>For cloning *col-20* cDNA	attcgatatcaagcttccaccttgacgg<br>ctatcatt	cggtatcgataagcttcgtttggtc<br>cattgtctcct
pHMZ173[*bli-6* RNAi]<br>For cloning *bli-6* cDNA	attcgatatcaagcttcgcacgtgaag<br>aagggtatt	cggtatcgataagcttggtcctgg<br>atttccatcaga
pHMZ174[*rol-1* RNAi]<br>For cloning *rol-1* cDNA	attcgatatcaagcttaaaaccaccacg<br>accagaag	cggtatcgataagcttacgtggtc<br>cttgtcttccac
pHMZ175[*lon-3* RNAi]<br>For cloning *lon-3* cDNA	attcgatatcaagcttcttcgaatcgtat<br>ccgtcgt	cggtatcgataagcttatcctgga<br>actccgacctct
pHMZ67[P*dpy-2*::*dpy-2*::mCherry]<br>For cloning *dpy-2*	aggacccttggctagctagcagtatttc<br>tgaatttctctttca	ctcgaggactgctagctgtgtgaa<br>tttacgcaagtga
pHMZ11[*dpy-3*::mCherry]<br>For cloning *dpy-3*	aggacccttggctagctgtgaccctac<br>ggctcaaat	ctcgaggactgctagcggtggtg<br>gacaatgatcaca
pHMZ68[*dpy-4*::mCherry]<br>For cloning *dpy-4*	aggacccttggctagctccaatgaaga<br>ttactcactattgttt	ctcgaggactgctagcaagattcc<br>tccgtccaaagc
pHMZ69[*dpy-5*::mCherry]<br>For cloning *dpy-5*	aggacccttggctagcctgttagcccg<br>gaaaaatca	ctcgaggactgctagccgtctgcg<br>ctttctctctg
pHMZ12[*dpy-17*::mCherry]<br>For cloning *dpy-17*	aggacccttggctagcggaggtgattc<br>tcgtggtgt	ctcgaggactgctagctttctgtat<br>ccttggtttccagag
pHMZ96[P*mup-4*::*mup-4* (extracellular domain deletion)::GFP)]<br>For cloning *mup-4* promoter	ggaggacccttggagggtaccattaat<br>tccgcagcaacgaa	aggatgagtggcagattcgaggt<br>ctgccgatgttgatagg
pHMZ96 [P*mup-4*::*mup-4* (extracellular domain deletion)::GFP]<br>For cloning *mup-4* ORF without extracellular domain	tcgaatctgccactcatcct	ctcatttttctaccggtacctctgct<br>ttggtcgttgtca

Gene	QPCR Forward primer (5'-> 3')	QPCR Reverse primer (5'-> 3')
nlp-29	tccttctcgcctgcttca	ctttccccatcctccataca
cnc-2	tgatgggaggttatggagga	gagcattccaaggagtccag
dpy-4	ttaaggcttaccgcttcgtc	agagttccacacgtccttgg
dpy-5	ctgagcaatacgttcgtgga	tccagttggtccgttctttc
dpy-7	tgctcagcattcagttggac	cattgttggttgtcggattg
dpy-9	gacttccaggaccacaagga	ccgatggatacgactctggt
dpy-17	catcgaacaccccaactctt	gcatcttctccgtcaactcc
